# =Observations on the Irritability of Vegetables, with an Account of Some Experiments Made by M. Hubert, to Determine the Heat Evolved by the Arum Cordifolium during the Process of Fecundation

**Published:** 1808-12

**Authors:** 


### *Observations on the Irritability of Vegetables, with an Account of some Experiments made by M.* HUBERT, *to determine the Heat evolved by the Arum Cordifolium during the Process of Fecundation. By Dr.* HALL.

THE capacity which animal bodies possess of being excited into action by the application of stimuli, has, it is well known, been denominated by philosophers the principle of excitability, or as others chuse to term it, irritability. Numerous experiments incontestibly demonstrate that vegetables, like animals, are endowed with a similar principle, on which depend not only the absorbing power and the ascent of the sap, but also the motion or circulation of the juices in their vessels. It is beyond a doubt, that fluids are absorbed and propelled by vegetables, so long as their vitality remains, and that, as soon as this principle is destroyed, the circulation immediately ceases. It also appears, that irritability exists more particularly in some parts of vegetables than in others, and that it likewise varies under different circumstances, and at different periods of their growth. This property, which is peculiar to both animal and vegetable bodies, had long attracted the attention of physiologists, and various hypotheses were at different times invented to explain it. But it was not till towards the end of the eighteenth century, when the invention of thermometers enabled them accurately to measure the heat of bodies, that any precise knowledge was obtained on this subject.

As your Journal embraces not only medical but physical subjects, I flatter myself that the following account of some curious and important experiments, made by M. Hubert, an intelligent naturalist in the Isle of France, and the celebrated traveller M. St. Vincent, respecting the heat evolved by some plants during the process of fecundation, cannot fail to prove highly acceptable to every one interested in the investigation of the laws and operations of Nature, and in tracing the analogy that subsists between the two great classes of organized bodies.

The *arum*, which was made the subject of these experiments, is a native of Madagascar, and of some other African Islands. It is a new species, termed by M. St. Vincent *arum cordifolium*, and of which he gives the following description. *Arum cordifolium caulescens, rectum, foliis ovato-cordatis, subundulatis, basi emarginatis.* Its flowers exhale a very strong odour, which far from being disa-

 greeable, greeable, like that of its congeneric species, is on the contrary rather pleasant.

The root of this plant is very thick, and penetrates deep into the soil. Its stem is strong, upright, and about four or five inches in diameter. The leaves are disposed in the form of a ridge, and fall off successively as they become old, leaving the mark of their petioles on the stem: they are cordate, oval, of a fine green colour, slightly undulated, very broad, and frequently a foot and a half in length; their nerves are pale and distinct, their petioles or footstalks are very long, round towards their upper extremity very broad, and deeply furrowed at their insertion, where they are semi-amplexicaul, and transparent at their edges. The flowers, which are upright, and supported on short pedicels, issue from their bases. The spathe is greenish externally, and yellowish within, as well as the rest of the organs of fructification. The *arum cordifolium* differs from the *arum arboreum*, to which it bears some resemblance in the stem, which is thicker, and not so branchy; in the colour of its leaves, which are not of so deep a green, and in their form, which are not sagittate or arrow-shaped; in its spadix, which is not reticulated; and in the base of its spathe, which is not of a deep red. It likewise differs from the *arum seguinum*, L. by its larger dimensions; by the leaves, which are emarginate or notched at the base, and finally, because it is not furnished with a nectary.

M. Hubert having observed, that the flowers of the *arum* yielded a stronger heat about sun-rise than at any other period, tied five spadices, which had been evolved during the night, round a thermometer. This number was necessary to cover the whole tube of the instrument. At sun-rise, the thermometer of comparison stood at 19°; it remained the same at six o'clock, while that of the experiment rose to 44°. At eight o'clock in the morning, the thermometer of comparison was at 21°; that of the experiment had fallen to 42°, and the heat of the spadices diminishing continually, at nine o'clock at night it was no more than 28°, while the first remained at 21°.

The next day, at nine in morning, the thermometer of experiment followed the ordinary course. M. Hubert repeated these trials several times, with nearly the same results. The mercury rose to 45°, when he surrounded it with very fine large spadices, but only reached 42° when he employed very small ones.

He succeeded in disposing twelve flowers of the *arum* round round the thermometer before the rising of the sun. The *maximum* of the heat was 49 ½°.

This indefatigable naturalist next divided five spadices longitudinally, and applied them against the thermometer in the direction of their sections. The *maximum* of the heat was 42°. This experiment, several times repeated, having led M. Hubert to suppose that the medulla, or pith of the spadices also gave out heat, he contrived to obtain that of a spadix, after having cut it at two inches from its point, by means of a small tin tube four lines in diameter, in order to plunge into it the elongated bulb of a thermometer. Twenty minutes after sun-rise, the mercury was at 39° which was the *maximum* of the heat; the thermometer of comparison stood at 17°. The heat of the mutilated spadix, observed the same periods as that of the most healthy spadices; it began to diminish at seven o'clock in the morning, and finished in the following night. He frequently repeated this experiment; and according to the size of the spadices, and the greater or less mutilation they had undergone in losing their medulla, he obtained 36, 37, or 38 degrees of heat.

The experiments above related were repeated alternately in a dry room, and under the shade of thick and humid trees, without the difference of the place occasioning any sensible variation in the results.

Having hitherto made his trials with the cut spadices, he next determined to repeat them on the plant itself. Having with this view placed his thermometer in a spathe, before sun-rise he obtained 38°, but sometimes only 36 or 37; the heat always ceased on the following night.

After having cut the extremities from six spadices, he tied the male parts alone round the thermometer. The *maximum* was but 41°; the moment of this *maximum* occurred about half an hour after sun-rise. The heat continued much longer; for the next morning, about day-break, the thermometer stood at 30°, and at nine o'clock at night it marked 24°, when that of comparison was at 18° only.

Six female parts of the flowers of the *arum* raised the thermometer to 28°, frequently even to 30°, He took care to make the thermometer touch the *ovaria*, and to deprive them of that part of the spathe which incloses them, the upper part of which withers, and falls off a few days after the heat has taken place.

Having reflected, that the heat which he conceived he had observed in the medulla of the spadices, might probably have only arisen from their exterior surface, in order der to ascertain this circumstance, M. Hubert made the following experiments.

With a very sharp knife he removed all the surface of four spadices, without touching the medulla. He then fastened these four medullæ round a thermometer, which at sun-rise stood at 17°. During twenty-four hours, no sign of heat appeared, and the uncovered spadices withered towards the middle of the day. At the same time that he made this experiment with the medulla of the four spadices, he surrounded the bulb of another thermometer with the surfaces of these spadices, the heat of which raised the mercury to 39°. This experiment, which was several times repeated, convinced him that this singular faculty is developed in the exterior surface of the spadices, and within the thickness of one line at most.

According to M. Hubert, there is reason to believe, that the heat indicated by the thermometer would have been greater, if the spadices could have been brought into contact with every part of the bulb, or of the tube of the instrument.

The following are some other experiments on the heat of the flowers of the *arum:* the spathe tied against the spadix during the fecundating process, withered as if it had been steeped in warm water.

Three spadices when evolving heat, having been placed in a caper-bottle, it soon became dim; in half an hour its inner surface was covered with drops of water; in an hour more there was an inch at the bottom of the bottle. He obtained a cubic inch of it in twenty-four hours. This water, which was without colour, and almost inodorous, dissolved soap very readily.

In the evening he cut five spadices, the spathes of which shewed that they would open during the night; and after having fastened them round a thermometer, exactly as in his first experiment, he put their pedicels into water. At ten o'clock at night, the thermometer of experiment was one degree higher than that of comparison; the *maximum* of heat was 34° at sun-rise, instead of 44° or 45°, which the spadices furnished when cut only an hour before sunrise, and when these spathes had opened naturally. During the remainder of the day, the thermometer remained at 33° and 32°. On the following day, after the usual hour of the *maximum* of the heat, the thermometer was still two degrees above that of comparison.

Flowers cut thirty hours before their developement, opened slowly; their spathes separated one-half less from their spadices, and their heat did not raise the thermomerter ter above 25°. In general, the spadices, which were mutilated some time before the developement of their heat, gave out much less; and a colourless fluid escaped from the divided portions, which is not the case when the heat has been previously evolved. This state of the spadix occurs only once, and its heat generally continues twenty-four hours.

The following experiments, which were undertaken with the view of ascertaining whether it be possible to augment, diminish, or wholly suspend, the heat evolved by the flowers of the *arum*, during the process of fecundation, must we conceive, prove highly interesting to those philosophers who imagine that life is the mere result of the action of certain agents on our orginization.

M. Hubert covered with a cloth dipped in olive oil, a fine spadix before sun-rise; but scarcely was the heat perceivable, when it again almost immediately disappeared, and at the usual hour of the *maximum*, was not distinguishable; by suffering the covering to remain during the rest of the day, the thermometer of experiment and that of comparison followed a similar course. Tallow and grease produced the same effect. On plunging spadices, when at their highest temperature, into cold water, their heat quickly disappeared; but was again renewed on their being withdrawn in the space of twenty-five to thirty minutes.

By immersing spadices at their ordinary temperature into cold water before sun-rise, and allowing them to remain in it till noon, the heat was evolved, even in this situation, and raised the thermometer to 37° or 38°, in half an hour.

After leaving the spadices twelve hours in water, at the end of that period they still raised the thermometer to 28°, and sometimes even to 30°, immediately on being withdrawn. In this experiment M. Hubert observes.—1st, That if the spadices be immersed in the water after the hour of the *maximum* of the heat, their temperature will not be so high on being taken out. 2d, That if any of the extremities of the spadices be allowed to swim on the surface of the water, the supernatant portions do not experience any diminution of temperature, but, on the contrary, are of the same degree of heat as if the rest of the flower had been exposed to the open air. And, lastly, that on the immersed portions of the spadices being withdrawn, and the suspended heat suffered to re-appear, the upper extremities in which the heat was evolved in the open air, did not yield yield more than the portions which had been kept under water.

Spadices kept twenty-fours under water, did not raise the thermometer more than 2° or 3° above the temperature of the surrounding atmosphere.

Spadices immersed nine minutes in water, previously heated to 41°, on being withdrawn raised the thermometer to 34°; but they withered on immersion in water of a higher temperature.

M. Hubert next placed a thermometer, for a quarter of an hour, in the middle of a spadix immersed in spirits of wine. On being withdrawn, the thermometer fell 4° below the temperature of the atmosphere; which, in his opinion, was attributable to the cold produced by evaporation, as in a short time it ascended from 35° to 39°. In this experiment the spirits of wine must be prevented from entering the upper part of the spadix, otherwise the medulla and external part of the flower will become dry in a short time.

He coated some spadices three times with essential oil of cloves; and afterwards placed three on one thermometer and one on another. The first indicated 30° of heat and the second 35°. The lower degree of heat in the first case, most probably, arose from the essential oil not being completely evaporated, and the concrete portion which remained producing a similar effect with the fat oil employed in a former experiment.

Spadices plunged for a short time into highly concentrated vinegar, recovered their heat on the evaporation of the fluid.

A spadix, which had been five times wetted with vitriolic ether by means of a feather, raised the thermometer to 38°.

When covered with honey, the evolution of the heat was suspended in the spadices for almost an hour.

Spadices deprived of heat, by being wrapt up in several folds of a black or white stuff, indicated the same degree of heat, and at the same periods, as when uncovered.

Having tied up, as closely as possible, in a pig's bladder, from which the air had been previously expelled, a thermometer surrounded by five spadices, it indicated only 30°; but on being withdrawn from the bladder, at 8 'clock in the morning, it almost immediately rose to 45°.

A spadix covered with starch prepared from the powder of the manioc, gave out no heat till its covering dried and fell off in small portions.

Having

Having formed paper tubes, of a size merely sufficient to contain a spadix, in which a thermometer was previously placed, he closed them, so as to prevent the air finding its way into them along the instrument. The heat was perceptible to the hand on touching the external surface of the tube, and the thermometer indicated 37°.

Four spadices, placed in a similar apparatus, raised the thermometer to 43.

In these two last experiments no water was produced, as in the caper-flask; the paper tubes on examination were perfectly dry. In another experiment he coated the tubes with very thick starch, renewing it every half-hour. The heat present in the spadices at sun-rise was destroyed; and the thermometers, during the whole day, continued to indicate the same degree as one exposed to the influence of the atmosphere. On withdrawing the spadices from the tubes to which they were luted, the heat was re-evolved. A difference in the colour of the tubes produced no difference in the results.

When only one coating of starch was employed, the heat of the spadices became perceptible on its drying.

A spadix introduced into a phial of Cologne water hermetically sealed, gave out no heat; in a pint bottle of the same water, on the contrary, the heat was very perceptible; arising probably from the greater quantity of air contained in the larger volume of water.

In carbonic acid gas, in the air contained in the joints of the bamboo, and in the inflammable air of marshes, the spadices preserved their heat.

Having allowed several spadices to remain in a caper-flask well closed during five hours, a chicken which was introduced into it at the end of that time was immediately suffocated, but soon recovered on being quickly withdrawn. A taper was afterwards extinguished in the same bottle.

It would be needless here to enter into a detail of various other experiments made by M. Hubert, as they are wholly unconnected with the temperature of the spadices of the *arum cordifolium*; it were much however to be wished that the culture of this plant could be extended to Europe, and that some of our intelligent naturalists would employ themselves in examining the phænomenon which takes place during the process of its fecundation.

The *arum cordifolium* continues in flower from May to February; but it is during the latter of these months that the flowers are most numerous, and attain the highest degree of beauty and perfection.

 In

In 1777, M. Lamark observed, that the spadices of what he terms the *arum italicum** produced a very sensible degree of heat. "When," says he, "the expanded catkins of this shrub have acquired a certain state of developement or of perfection, perhaps at the period when the process of fecundation is going forward, their temperature is so much increased as to convey when handled a sensation of burning heat, far above that of other bodies exposed to the atmosphere. That the heat evolved by these catkins during the state we have already mentioned, is peculiar to them, and produced in their substance, is sufficiently evinced by the following circumstances. Of several catkins composing a tuft which was carefully examined, only one or two of them at a time exhibited this increase of temperature; while the others remained of the same degree of heat as the surrounding atmosphere, till, in their turn, attaining the necessary degree of perfection, they successively displayed the same remarkable phenomenon; this increased temperature continues only a few hours.

From the foregoing experiments it evidently follows, that plants possess a peculiar heat during the whole course of their vegetation, but that this heat, which doubtless depends on their vital action, and which apparently acquires different degrees of intensity, either in certain parts or at particular periods of their growth, is probably so feeble in the greater number of vegetables as to elude notice. It is besides probable, that many other plants would be found to display similar phenomena, at least in those parts destined to their reproduction, if they were examined with sufficient care and attention. Finally, it cannot be doubted that other species of *arums*, as well as all the plants appertaining to this family, are subject to an increase of temperature, under similar circumstances, though in a degree more or less intense in proportion to the thickness of the catkins. It appears truly astonishing, that among the learned men who since this period have written on vegetable physiology, the generation of plants, and the irritability of their sexual organs, not one of them appears to have bestowed sufficient attention to this important discovery of M. de Lamark; though such a circumstance is certainly deserving of the most accurate investigation.

Perhaps this heat exists in the anthers of all plants; but being

---

* *Arum Italicum*, accaule, foliis hastato-sagittatis, auriculatis, divaricatis, spadice cylindrico luteolo. Encyc. Meth.

being evolved only in proportion to their size, it may not be perceptible by our senses in the smaller vegetables. Should future and more accurate experiments, however, confirm the truth of this conjecture, it may perhaps explain the peculiar motion of certain stamens, the manner in which the eruption of the fecundating pollen is effected, and several other phenomena, with the causes of which we are still unacquainted.

It is well known, that snow melts more rapidly on meadows than on roads, or other grounds destitute of vegetation. Now as gramineous plants are frequently in flower during winter, may not the more rapid liquefaction of the snow be explained from the heat evolved by the anthers of these plants? Besides, in their natural relations, the grasses are sufficiently allied to the *arum* to warrant such a supposition.

From the experiments of M. Hubert, it would seem, that the mutilation of the spadices does not prevent the development of their heat; that this evolution of heat is carried on independently of the presence of light, but that the contact of atmospheric air is necessary to its production.

This gentleman having communicated the above experiments, as well as others, he had made on the *arum esculentum*, L. to M. St. Vincent, during his visit to the Isle of France, he justly conceived, that the spadices of every species of *arum* must possess a similar calorific property. In order to ascertain this, "I repeated," says M. St. Vincent, "several experiments which M. Hubert had made on the *arum esculentum*, L. in which he had scarcely discovered a perceptible degree of heat. In my experiment, on the contrary, a single flower, at the same hour, and under the same circumstances as the one he examined, raised the thermometer six degrees above the temperature of the surrounding atmosphere."

With respect to the time at which the heat of the spadices of the *arum* is evolved, I conceive it must depend on the same circumstances as the expansion of the corols in vegetables, which flower and lose their flowers at a particular and fixed period. It is not improbable, that the blossoms of all plants expand in consequence of the heat of the stamens acting on the irritability of the petals.

While reflecting on the consequences which might result from the heat of the spadices of the *arum*, I observed, in the cool of a fine morning, a great number of bees covering the male catkins of the *Pandanus utilis*, so as to exclude them wholly from the view. As these catkins are formed formed of a collection of stamens, I could not doubt that the bees frequented them with the twofold intention of collecting honey, while at the same time they were cherished by their heat. Too indolent, however, at the time to ascertain the truth of this conjecture, I returned on the following day, to examine a thermometer I had placed on some catkins which had blown during the night; but I was disappointed in the results I expected, owing to the sun being too far above the horizon.

In a short time after, I convinced myself in another manner, that a sensible degree of heat is evolved not only in the anthers of the *Pandanus utilis*, but likewise in those of different species of canes. With this intention I cut some thin slices of a substance which readily liquefies, such as butter of cocoa, and applied them along several stamens, in such a manner, that on melting it sunk into the substance of those portions of the plant with which it was in contact.

It were much to be wished, that Physiologists would pursue a course of experiments which promises such beneficial results, especially if conducted with the same care and discernment as those made by the celebrated Naturalists above-mentioned. R. H.

*Clifford's Inn, Nov.* 8, 1808.

