# Corrigenda

**Published:** 1808-12

**Authors:** 


### CORRIGENDA.

Line 16, page 499, after the word growth, read, "intimately connected with the principle of irritability is the power of evolving heat."

Line ditto, page ditto, for this property, which is peculiar, read, "this property, which is *also* peculiar.

